# Fat-intra-body communication system using flexible wearable antennas with human and torso phantom validation for biomedical applications

**DOI:** 10.1038/s41598-026-55490-0

**Published:** 2026-06-02

**Authors:** Bappaditya Mandal, Tarakeswar Shaw, Pramod K. B. Rangaiah, Laya Joseph, Arvind Selvan Chezhian, Maria Mani, Roger L. Karlsson, Robin Augustine

**Affiliations:** 1 Microwaves in Medical Engineering Group, Division of Integrated Smart Systems Technology, Department of Electrical Engineering, Uppsala University, 75121 Uppsala, Sweden; 2 Department of Electronics and Communication Engineering, Birla Institute of Technology (BIT), Mesra, Ranchi, 835215 India; 3 Department of Surgery, Section of Plastic and Maxillofacial Surgery, Uppsala University, Uppsala, Sweden; 4 Department of Plastic and Reconstructive Surgery, Uppsala University Hospital, Uppsala, Sweden

**Keywords:** Biological techniques, Biotechnology, Engineering, Medical research

## Abstract

This article introduces a biocompatible, non-invasive intra-body communication (IBC) system that employs a U-shaped, flexible wearable antenna specifically designed for fat-intra-body communication (Fat-IBC) applications. The proposed Fat-IBC system is engineered to function within the industrial, scientific, and medical (ISM) frequency range of 2.40–2.48 GHz. The U-shaped wearable antenna is encased in a biocompatible polydimethylsiloxane (PDMS) coating layer, enhancing durability and safety, making it suitable for continuous use in medical contexts. The U-shaped Fat-IBC antenna leverages the low-loss property of adipose (fat) tissue, positioned between skin and muscle layers, to facilitate efficient signal propagation across the body. The antenna is optimized for transmission through a three-layer tissue model (skin, fat, and muscle), leveraging the favorable transmission properties of adipose tissue to minimize signal loss. To assess performance, detailed numerical simulations and experimental validations were conducted using three-layer tissue models, torso phantoms (obese and athletic), and human volunteer trials. Ethical approvals were obtained for human testing, ensuring compliance with biomedical research standards. The performance of the U-shaped Fat-IBC antenna was benchmarked against a standard Bluetooth low energy (BLE) chip antenna under controlled conditions, with measurements taken in both front-to-back and side-to-side orientations across various body types. Experimental results demonstrated the superior performance of the U-shaped Fat-IBC antenna over BLE, specifically in terms of signal stability and transmission efficiency across body tissue, especially within adipose layers. Specific Absorption Rate (SAR) analysis revealed a peak value of 0.3061 W/kg, which is well within the IEEE safety limits, confirming the system’s suitability for biomedical applications. This Fat-IBC platform demonstrates the feasibility of fat tissue as a viable medium for intra-body networks, offering a reliable, power-efficient solution for real-time medical telemetry and patient monitoring, with promising implications for broader health applications.

## Introduction

Intra-body communication (IBC) has rapidly emerged as a transformative technology in medical and healthcare systems, revolutionizing how devices implanted within or worn on the human body communicate with each other^1,2^. By leveraging human body tissues as the transmission medium, IBC offers key advantages, including ultra-low power, reduced external electromagnetic interference, and enhanced data security, which is critical for sensitive medical information^3^. These attributes make IBC particularly advantageous for the continuous management of chronic diseases such as cardiovascular disorders, diabetes, and other noncommunicable diseases (NCDs) that impact millions worldwide^4–7^. The unique ability of IBC systems to provide secure, real-time data transmission from critical medical devices such as pacemakers, cochlear implants, and continuous glucose monitors significantly improves the monitoring and treatment of chronic conditions^8,9^. The capability underscores the potential of IBC in addressing the growing global burden of chronic diseases, advancing the field of implantable and wearable medical technologies.

In the realm of intra-body communication (IBC), various approaches have been explored and documented in the literature^10^. These methodologies are broadly categorized into galvanic coupling^11^, capacitive coupling^12,13^, resonant coupling^14^, ultrasound^15,16^, and radio frequency (RF)-based communication^17^. Each IBC technique exhibits unique characteristics, enabling its adaptability across diverse medical applications, particularly in closed-loop monitoring systems where real-time remote adjustments to treatments are possible. Galvanic coupling, for instance, employs a low-intensity current transmitted through electrodes, making it effective for localized, low-power communication via conductive tissues such as muscle and skin. However, it faces limitations in penetrating non-conductive tissues, restricting its operational range^11^. On the other hand, capacitive coupling utilizes displacement currents via capacitive plates, offering a non-invasive option suitable for wearable devices^12^. Despite its suitability for non-invasive applications, it is prone to signal attenuation and interference. Ultrasound and resonant coupling techniques promise higher data transmission rates, though their effective range is often limited to short-distance applications. Further, different frequency bands are also considered to construct a medical IBC system, such as lower frequencies (typically below 100 MHz) for the human body communication (HBC)^18^, medical implant communication service (MICS, 402-405 MHz band)^19^, and ultra-wideband (UWB) for high data rates^20^. Conversely, RF communication stands out for long-range data transmission, making it ideal for connecting multiple implants or external devices. However, it demands higher power and carries the risk of tissue heating due to electromagnetic wave propagation^17^. The selection of an appropriate IBC technique and operating frequency depends on the specific medical application, the device location, and the required communication distance. Despite the challenges, IBC technologies remain integral to continuous monitoring and secure interaction between body-worn or implanted devices^21^. The ongoing research aims to advance IBC systems, focusing on higher bandwidth, extended range, high data transfer rate, and improved adaptability to dynamic environments.

**Fig. 1:**
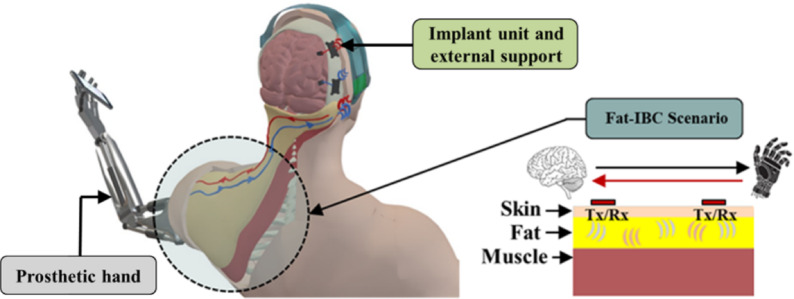
Conceptual schematic of a bidirectional Fat-IBC system for bio-telemetry, showing signal transmission and reception through adipose tissue as a propagation medium, with integrated transmitter (Tx) and receiver (Rx) pathways for in-body communication.

To overcome the limitations of conventional intra-body communication (IBC) methods, such as restricted bandwidth and low data rates^11,12,14,15,17^, a novel fat intra-body communication (Fat-IBC) approach has been introduced through the EU H2020 FET Open project B-CRATOS (965044)-“Wireless Brain-Connect Interface TO machineS”^22^. This groundbreaking project seeks to transform the lives of individuals with paralysis or limb loss by developing a bidirectional neural interface system, facilitating direct communication between brain implants and advanced prosthetic devices. The core innovation of the Fat-IBC system lies in its utilization of the dielectric properties of fat tissue, which has lower permittivity and conductivity, resulting in reduced signal loss compared to muscle and skin tissues^23,24^. These properties are particularly advantageous for electromagnetic wave propagation at microwave frequencies, allowing for more efficient signal transmission with minimal attenuation. As a result, Fat-IBC offers improved communication reliability and data transfer rates, making it a more effective solution for complex biomedical applications. The conceptual representation of the two-way data communication with the Fat-IBC-based approach is presented in Fig. 1. The conceptual representation highlights a healthcare monitoring framework in which multiple wearable and implantable devices seamlessly exchange data using the human body as the signal propagation medium. This approach supports coordinated, low-power communication within the body, enabling continuous, reliable, and minimally intrusive health monitoring.

To demonstrate the potential of Fat-IBC, various methodologies have been explored in the literature^25–28^. A notable approach characterizes adipose tissue and muscle as transmission channels for IBC in the 1.7 to 2.6 GHz frequency range, providing critical information on signal propagation within these biological media^25^. Experimental validation of data packet transmission through fat tissue has been conducted using both phantom models and ex-vivo measurements, confirming the feasibility of this method for intra-body network applications^26^. Furthermore, the impact of blood vessels on the Fat-IBC system was rigorously examined through numerical simulations and ex vivo models, particularly at the 2.45 GHz frequency^27^. This analysis is crucial in understanding the interaction of electromagnetic waves with biological structures, further optimizing signal performance for real-world applications. Recently, leveraging the Fat-IBC approach has enabled the achievement of a high data rate of 92 Mb/s using low-cost WLAN hardware, underscoring the system’s capability to facilitate efficient communication even with commercially available technology^28^. Most recently, wearable antennas incorporated with ferrite materials and copper shielding have been developed to effectively suppress surface wave propagation over the human body, thereby enhancing the overall performance of Fat-IBC systems^29,30^. Most recently, a smart shield wearable antenna has been presented^31^ for efficient fat-intra-body connectivity. These findings suggest that Fat-IBC offers significant advantages in high-data-rate communication, particularly in complex biological environments where muscle obstructions and other tissues often hinder signal propagation. However, there remains a growing demand for the development of simple, planar, biocompatible, and non-invasive wearable systems to fully utilize the potential of Fat-IBC for advanced biomedical devices.

**Fig. 2:**
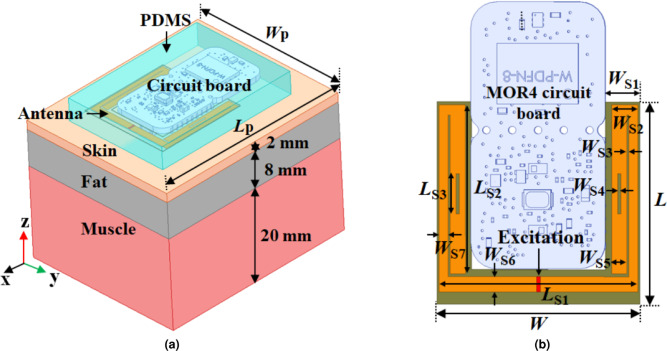
(**a**) Wearable U-shaped Fat-IBC antenna placed over three layer (skin, fat, and muscle) tissue model with circuit components and PDMS coating. (**b**) Top view of the U-shaped antenna along with MOR4 circuit board.

**Table 1:** Dielectric property of the human tissue used during the simulations at 2.45 GHz.

Human tissue and thickness	Relative permittivity	Conductivity (S/m)	Loss tangent
Skin (2 mm)	38.08	1.44	0.28
Fat (8 mm)	5.28	0.10	0.145
Muscle (20 mm)	52.8	1.71	0.24

This paper presents a new flexible, planar, and biocompatible wearable antenna-based system that operates in a non-invasive manner. The design utilizes the Fat-IBC technique to enable efficient and reliable high-speed data transmission. To achieve this, a flexible U-shaped wearable antenna is developed, operating at 2.45 GHz. The proposed system utilizes two identical antennas, coated with polydimethylsiloxane (PDMS), to avoid direct skin contact and enhance bio-compatibility. Numerical simulations and experimental validations were performed to analyze the performance of the proposed Fat-IBC system. The system’s effectiveness was assessed through transmission coefficients and signal strength measurements. Comprehensive numerical simulations and empirical measurements were performed to validate the performance of the proposed U-shaped flexible antenna system within a Fat-IBC framework. These experiments utilized realistic obese and athletic torso phantoms, alongside trials with human volunteers conducted under ethical approval. In this study, for the first time, a U-shaped flexible wearable compact antenna specifically designed and optimized for Fat-IBC applications, offering conformability to body curvature for continuous health monitoring that has not been addressed by prior Fat-IBC antenna designs^28–30^, which usually used rigid substrates. In addition, for the first time, the human volunteer trials have also been conducted to establish the concept of Fat-IBC system. To benchmark performance, a standard Bluetooth low energy (BLE) chip antenna was included in the study. The test involved positioning both U-shaped and BLE antennas in various configurations (front-to-back and side-to-side) on both torso phantoms and human volunteers with different body compositions. The results of this comparative analysis reveal the superior signal stability and efficiency of the proposed U-shaped antenna over the BLE, particularly in maintaining high transmission quality. Additionally, the specific absorption rate (SAR) analysis indicated a peak value of 0.3061 W/kg, which is within the IEEE regulatory limits. The proposed Fat-IBC platform establishes adipose tissue as an effective medium for intra-body communication networks, supporting reliable, low-power signal transmission ideal for real-time medical telemetry and continuous patient monitoring.

## Design of the compact flexible wearable antenna for fat-IBC system

In designing a Fat-IBC system, the antenna and transceiver play an important role in ensuring robust and efficient signal transmission through biological tissues. The antenna serves as the primary interface for high-speed intra-body communication, facilitating effective signal transmission and reception through adipose tissue. Its design and optimization require an in-depth understanding of the dielectric and electrical properties of human tissues at the target frequency, typically around 2.45 GHz, where signal propagation is enhanced within fat layers due to their low permittivity and reduced absorption characteristics^23,24^. Additionally, the transceiver system is designed to operate within strict regulatory limits on power output to prevent tissue heating while maintaining a high signal-to-noise ratio (SNR). Collectively, the antenna-transceiver configuration in a Fat-IBC system supports secure, high-speed, and low-power data transmission, essential for real-time biomedical applications in telemedicine and remote patient monitoring^25–27^. This approach underscores the necessity of integrating bio-compatibility and electromagnetic safety in wearable IBC devices for optimized patient care.

**Table 2:** Optimized dimensions of the U-shaped antenna.

Parameter	Value	Parameter	Value	Parameter	Value	Parameter	Value
*L*	20 mm	*W*	20 mm	$L_{s1}$	19.6 mm	$L_{s2}$	16.7 mm
$L_{s3}$	4 mm	$W_{s1}$	3.4 mm	$W_{s2}$	2.6 mm	$W_{s3}$	0.2 mm
$W_{s4}$	0.3 mm	$W_{s5}$	1.5 mm	$W_{s6}$	1.5 mm	$W_{s7}$	0.9 mm

Herein, a compact and flexible U-shaped portable wearable antenna with a MOR4 circuit board has been specifically designed to establish the Fat-IBC system. The MOR4 circuit board serves as the RF front-end interface between the U-shaped antenna and the measurement/communication system. It integrates impedance matching components and an SMA connector interface, enabling direct connection to measurement equipment (VNA) or transceiver modules. The MOR4 board was selected for its compact form factor and compatibility with the 2.45 GHz ISM band, making it suitable for integration into the wearable Fat-IBC system. The human body tissues exhibit unique electromagnetic characteristics that pose both challenges and opportunities for intra-body communication systems. The U-shaped antenna is designed on a three-layer flat human tissue model comprising skin, fat, and muscle, as illustrated in Fig.2a. In this study, a flat multilayer phantom is considered to use as an initial modeling approach to study electromagnetic propagation through biological tissues while avoiding the complexity of anatomical curvature and heterogeneity. This simplification improves computational efficiency and enables clear analysis of field interactions with individual layers such as skin, fat, and muscle. Such models are widely adopted in early-stage in-body and on-body communication studies for their analytical tractability and interpretability^25–30^. The dielectric properties of the skin, fat, and muscle layers were sourced from the Institute of Applied Physics “Nello Carrara” (IFAC) data at 2.45 GHz^32^ and are summarized in Table 1. The antenna has been designed on a flexible Rogers RT/duroid 6010 LM dielectric material with a thickness of 0.13 mm. To prevent direct body contact, the antenna is encapsulated in a polydimethylsiloxane (PDMS) coating, ensuring biocompatibility and user safety.

**Fig. 3:**
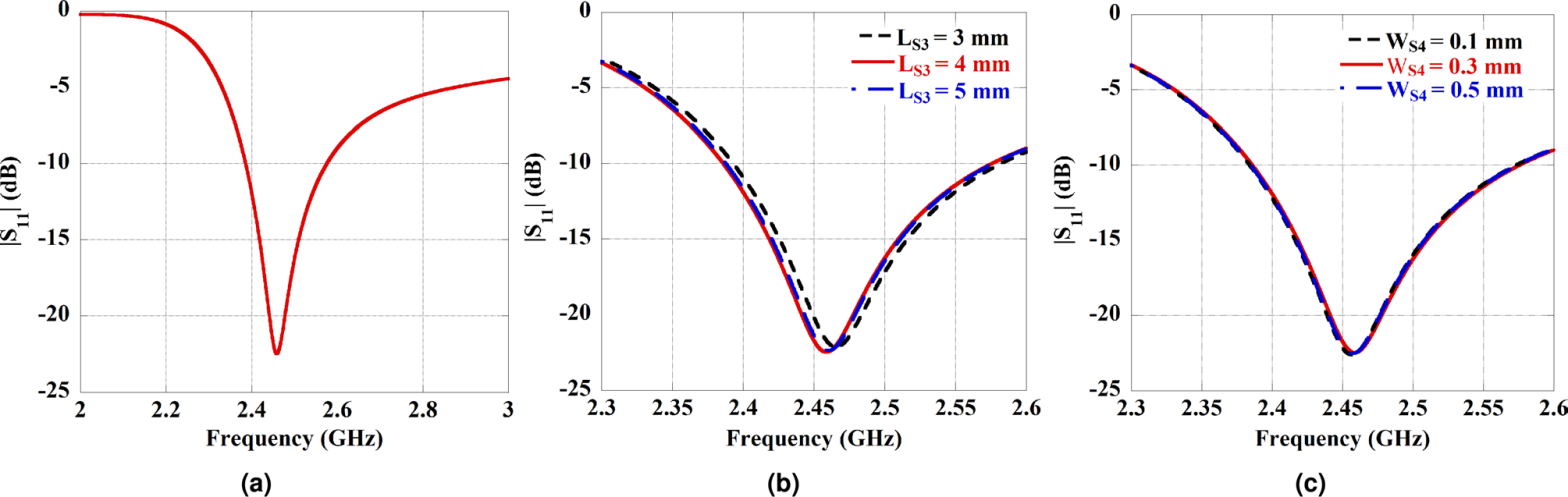
The Characteristics of the reflection coefficient ($|S_{11}|$) for the U-shaped antenna. (**a**) The $|S_{11}|$ for the proposed antenna configuration. The change in the reflection coefficient with the variation of slot, (**b**) Length ($L_{S3}$), and (**c**) Width ($W_{S4}$).

**Fig. 4:**
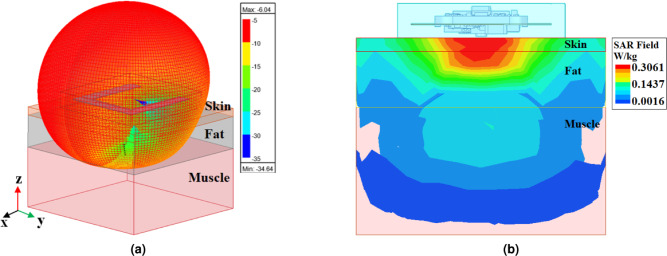
Characteristics of the wearable U-shaped antenna. (**a**) 3D polar plot of the radiation pattern. (**b**) The 1-g average SAR distribution in a three-layer human tissue model.

The U-shaped structure is tuned to operate within the 2.40–2.48 GHz industrial, scientific, and medical (ISM) band, with a resonance at 2.45 GHz. The 2.45 GHz ISM frequency band was selected due to several converging advantages: (i) it is license-free and globally available for medical and scientific use; (ii) it supports sufficient bandwidth for high-data-rate Fat-IBC applications^28–30^; (iii) it coincides with commercially available low-cost transceiver hardware (e.g., WLAN, BLE modules), facilitating practical system integration; and (iv) fat tissue exhibits favourably low attenuation at this frequency compared to skin and muscle, making it well-suited for fat-channel-based propagation^23,24^. Fig. 2b presents the simulation geometry of the U-shaped antenna with the MOR4 circuit board. Also, Table 2 highlights the optimized dimensions and design parameters of the proposed U-shaped antenna. The U-shaped topology was selected because it provides a compact, meandered current path that achieves resonance at 2.45 GHz within a small footprint, while maintaining a radiation pattern directed toward the body tissue layers. The U-shape also allows incorporation of tuning slots in the arms for fine frequency adjustment without increasing the antenna footprint. The characteristic of the reflection coefficient ($|S_{11}|$) for the proposed U-shaped configuration is shown in Fig. 3a, confirming the target operational frequency of 2.45 GHz. Moreover, a parametric study has been conducted to optimize the length ($L_{S3}$) and width ($W_{S4}$) of the slot on both sides of the U-shape arms to attain the desired operating frequency. The effect of slot length and width has also been included in Fig. 3b and 3c, respectively. Due to additional impedance loading with the change of length and width of the slot, a minor change in the reflection coefficient has been observed. In case of the proposed U-shaped antenna, $L_{S3}=4$ mm and $W_{S4}=0.3$ mm are considered as these values provide better matching along with the required operating frequency, 2.45 GHz. A detailed discussion about the effect of the parametric study of a U-shaped antenna has been presented in the reported work^33^. Also, -10 dB impedance bandwidth obtained from the proposed U-shaped antenna is 7.65%. Further, the 3D polar plot of the radiation characteristics is presented in Fig. 4a. The maximum boresight gain obtained from the 3D radiation plot of the proposed antenna is -6.04 dB at $\theta =0^0$ opposite to the human body model, while towards the body tissue model, –7.02 dB at $\theta =-180^0$, respectively. The differences in gain values are attributed due to the absorbing and lossy properties of human tissues. All simulations and electromagnetic analyses were performed using Ansys High-Frequency Structure Simulator (HFSS, version 2024 R1), a leading tool in 3D electromagnetic modeling. An adaptive mesh refinement strategy was employed, with a maximum delta-S convergence criterion of 0.02 applied over a minimum of six adaptive passes and fewer, depending on the simulation geometry. Radiation boundary conditions were assigned on all outer surfaces of the simulation domain (minimum $\lambda /4$ separation from the antenna structure), and a lumped-port excitation with 50 ohm reference impedance was applied at the antenna feed point. Tissue dielectric properties were assigned as frequency-dependent using the IFAC parametric model at 2.45 GHz.

The primary goal in developing Wireless Body Area Network (WBAN) or Intra-Body Area Network (IBAN) systems for biomedical applications is to ensure safety by limiting human exposure to electromagnetic (EM) fields^34–36^. This safety is quantified by the specific absorption rate (SAR), which measures the rate at which energy is absorbed by biological tissue under EM field exposure. According to the Federal Communications Commission (FCC) regulations, the maximum transmit power allowed within ISM frequency bands is 1 W to prevent harmful levels of exposure^37^. Furthermore, according to the IEEE safety guidelines, the SAR value should remain below 1.6 W/kg averaged over 1 gram of tissue to ensure safe operation for biomedical applications^38^. In this study, the SAR analysis of the U-shaped Fat-IBC antenna was performed in HFSS (version 2024 R1) with an excitation power of 1 W, corresponding to the maximum permissible transmit power under FCC regulations for the ISM band. The SAR was computed using the 1-gram tissue-averaging method in accordance with the IEEE and FCC Standards. The resulting peak 1-gram-averaged SAR was 0.3061 W/kg, well within the regulatory limit of 1.6 W/kg, confirming the safety of the proposed system under maximum-power operation. Additionally, to minimize direct exposure of electromagnetic energy to human tissues, the antenna was encapsulated within a biocompatible PDMS layer, providing insulation and compliance with medical standards.

### Analysis of data transmission using U-shaped fat-IBC antennas

**Fig. 5:**
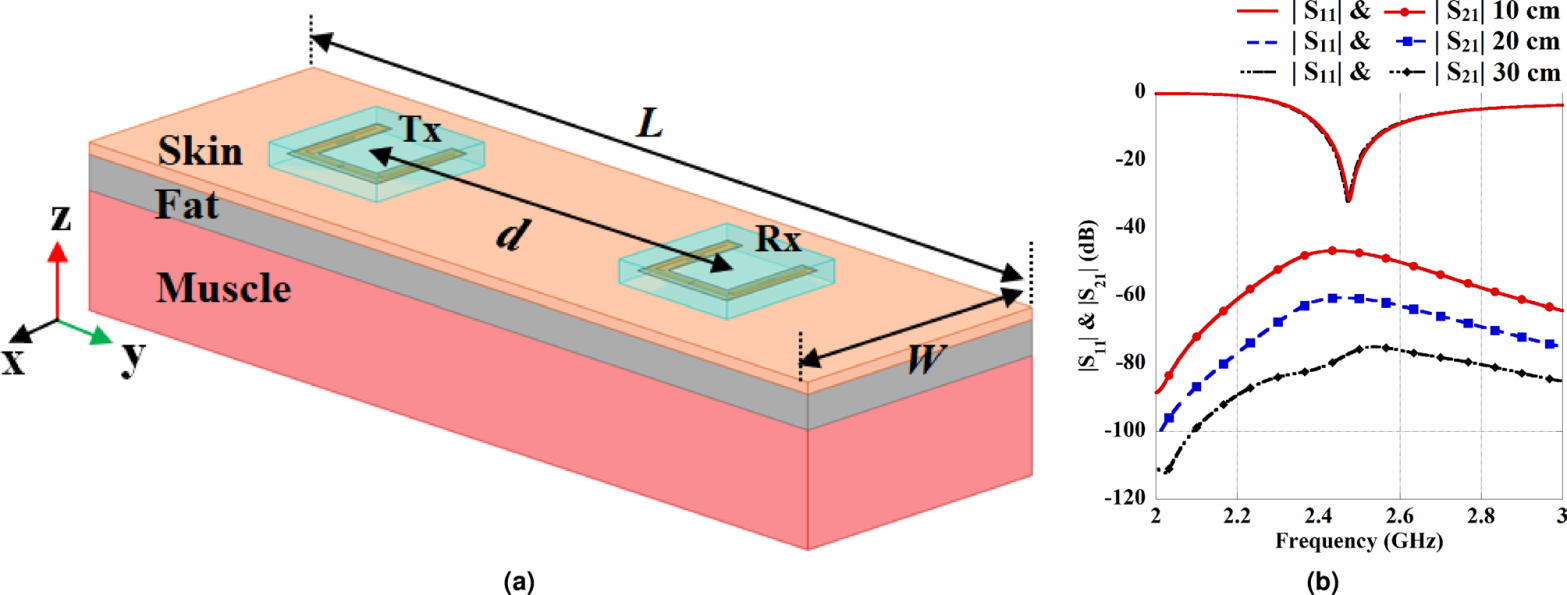
(**a**) Simulation setup for data transmission using U-shaped antennas for side-to-side (S2S) placement. (**b**) Characteristics of the transmission ($|S_{21}|$) and reflection coefficient ($|S_{11}|$) with respect to distance, *d*.

**Fig. 6:**
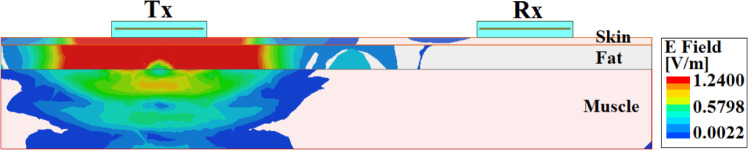
E-field distribution through three-layer tissue model.

This section presents a comprehensive simulation analysis for developing a Fat-IBC data transmission system using a multi-layered human tissue model. Two identical U-shaped flexible wearable antennas were implemented in the system to assess the impact on transmission performance. As illustrated in Fig. 5a, the layout of the system shows the placement of the Tx and Rx antennas on a three-layer model. The transfer distance, denoted as *d*, defines separation distance between center to center of the Tx and Rx antennas. The Fat-IBC system design takes advantage of the low-loss characteristics of the fat layer to allow efficient data transmission within this multilayered structure.

To evaluate the performance of the system, numerical simulations focused on key transmission parameters, specifically the transmission coefficients ($|S_{21}|$) and reflection ($|S_{11}|$). Fig. 5b provides the simulation results for $|S_{21}|$ and $|S_{11}|$ across transfer distances of 10, 20, and 30 cm. The results demonstrate that the working resonant frequencies within the ISM frequency range of 2.4 to 2.48 GHz are consistent across all tested distances, with minimal changes in impedance matching, validating the system’s adaptability over varying distances. As expected, the transmission amplitude $|S_{21}|$ decreases with increasing transfer distance due to natural signal attenuation. It can be perceived from Fig. 5b that the maximum transmission coefficients of –46.89, –60.60, and –74.96 dB for distances of 10, 20, and 30 cm have been obtained at resonance, respectively. Furthermore, the E-field distribution for a transfer distance of 10 cm at 2.45 GHz is presented in Fig. 6, which illustrates the propagation of the electromagnetic energy. The lateral energy flow is concentrated primarily through the fat layer, directing most of the energy towards the Rx antenna due to the fat’s lower attenuation properties relative to skin and muscle layers, as previously established^23,28–30^. This lateral propagation pattern represents the effectiveness of the fat layer as a low-loss transmission medium for intra-body communication.

**Fig. 7:**
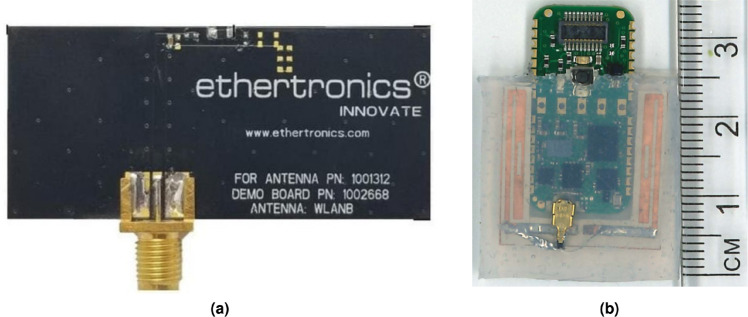
Antennas used for the comparison of data transmission measurements for the Fat-IBC system. (**a**) BLE chip antenna, and (**b**) proposed U-shaped antenna with MOR4 circuit.

**Fig. 8:**
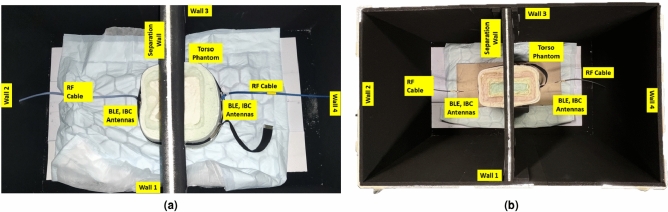
Measurement setups for transmission coefficient in semi-shield anechoic box with torso phantoms in front-to-back (F2B) antenna placement setup. (**a**) Obese abdominal torso, and (**b**) athletic thoracic torso phantoms.

**Fig. 9:**
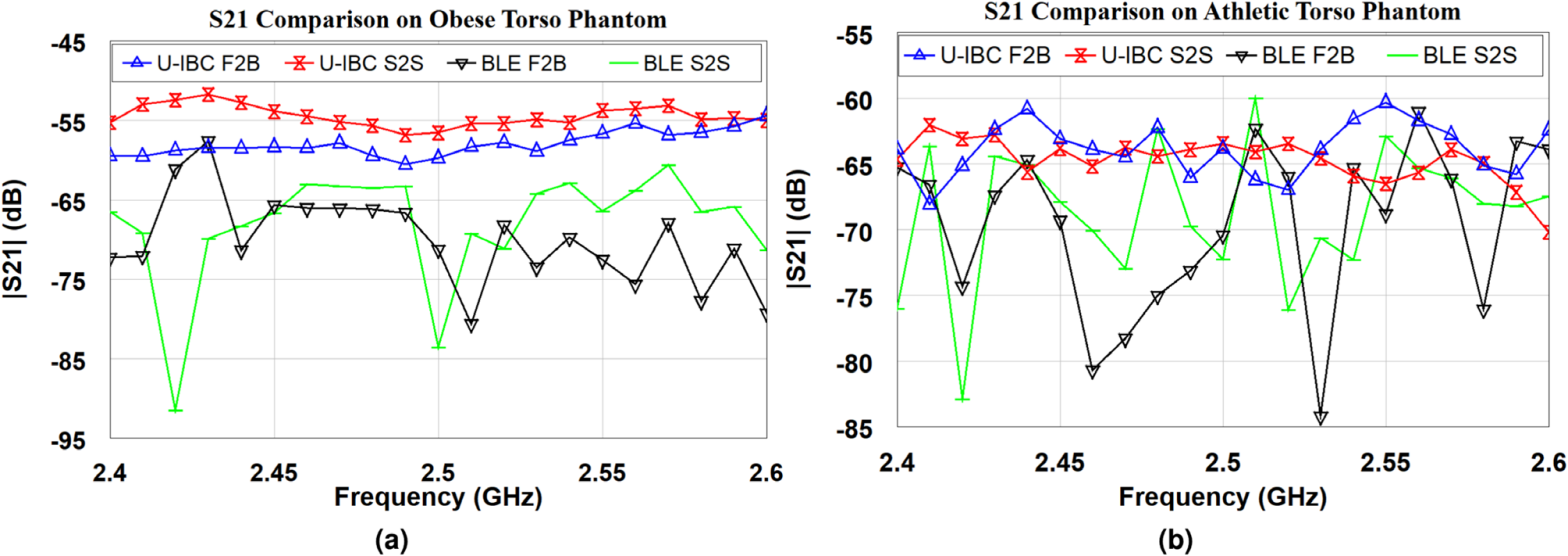
Measured transmission coefficient by using BLE and U-shaped Fat-IBC antennas by placing front-to-back (F2B) and side-to-side (S2S). (**a**) On obese torso, and (**b**) on athletic torso phantoms.

To validate the proposed Fat-IBC system for real-time biomedical applications, prototypes of the U-shaped wearable antenna were fabricated and subjected to rigorous testing. A primary design objective was to ensure biocompatibility and user safety. To achieve this, a PDMS coating was applied to the fabricated antenna prototype following the process discussed in the reported works^29,30^. This PDMS layer not only facilitates long-term wearability by offering a biocompatible interface but also ensures structural integrity and insulation from bodily fluids, essential for medical applications. A comparative study was conducted to evaluate the performance of the custom-designed Fat-IBC antenna against a commercially available conventional BLE antenna, a ceramic BLE chip antenna (0402 series BT/WLAN), widely adopted in embedded and healthcare systems, smart grids, and M2M communication^39^. The prototypes of the BLE and U-shaped Fat-IBC antennas are depicted in Fig. 7a and b, respectively. The BLE antenna serves as a benchmark for low-power communication; the U-shaped Fat-IBC antenna, designed specifically for IBC, leverages fat tissue’s low-loss characteristics to enhance signal propagation within the human body. Detailed measurements of transmission coefficients ($|S_{21}|$) and signal strength were conducted across torso phantoms and human volunteers. These tests provide valuable insights, confirming the Fat-IBC system’s efficiency and reliability for healthcare applications, including medical telemetry, through real-world emulation setups.

**Table 3:** Comparative study between the transmission performance of BLE and U-shaped antennas for torso measurements at 2.45 GHz.

Torso type	Antenna type	Antenna Placement	$|S_{21}|$(dB)	$\Delta |S_{21}|$ (dB)
Obese	BLE	Front-to-back	−65.69	–
Phantom	Antenna	Side-to-side	−66.66	–
Obese	U-shaped	Front-to-back	−57.83	+ 7.86
Phantom	Antenna	Side-to-side	−53.77	+ 12.89
Athletic	BLE	Front-to-back	−69.33	–
Phantom	Antenna	Side-to-side	−67.89	–
Athletic	U-shaped	Front-to-back	−62.57	+ 6.76
Phantom	Antenna	Side-to-side	−69.76	-−1.87

## Measurement results and discussion

The Fat-IBC concept prioritizes biocompatibility, signal optimization, and practical deployment for real-world biomedical applications. Accordingly, comparison testing was conducted in a custom small-scale anechoic chamber specifically designed for Fat-IBC measurements. The semi-shielded chamber, with dimensions 100 cm $\times$ 60 cm $\times$ 60 cm, features high-insertion-loss microwave absorbers (EA-LF500-24) lining the interior walls to minimize internal reflections, is shown in Fig. 8. Its exterior is covered with a 30 $\upmu$m thick aluminum sheet, blocking external electromagnetic interference and securing RF isolation, essential for maintaining measurement integrity by filtering out GSM, Wi-Fi, and Bluetooth signals. The chamber’s top panel serves as an access lid, facilitating the placement of phantoms and measuring devices. Small orifices were introduced for vector network analyzer (VNA) cable insertion, carefully sealed using shielding absorber sheets to prevent signal leakage. A 0.8 cm thick wooden partition, clad in absorber material on both sides, segregates the testing space within the box. The internal wooden partition, clad with microwave absorber material on both sides, serves two purposes: (i) it physically supports the torso phantom in an upright position within the chamber, and (ii) it provides electromagnetic isolation between the Tx and Rx sides of the measurement, ensuring that the measured transmission coefficient ($|S_{21}|$) represents signal propagation primarily through the phantom body (via the fat channel) rather than through free-space leakage paths around the phantom. Following the EN50147-1-1996 standards for shielding attenuation, this setup ensures effective RF isolation for the 9 kHz-40 GHz range. Additionally, the lower absorber partition, standing 20 cm high, can be adjusted based on phantom geometry, allowing optimized field distribution during measurements. Further, a comprehensive description of the semi-shielded chamber design and fabrication is available in previously reported work^40^.

**Fig. 10:**
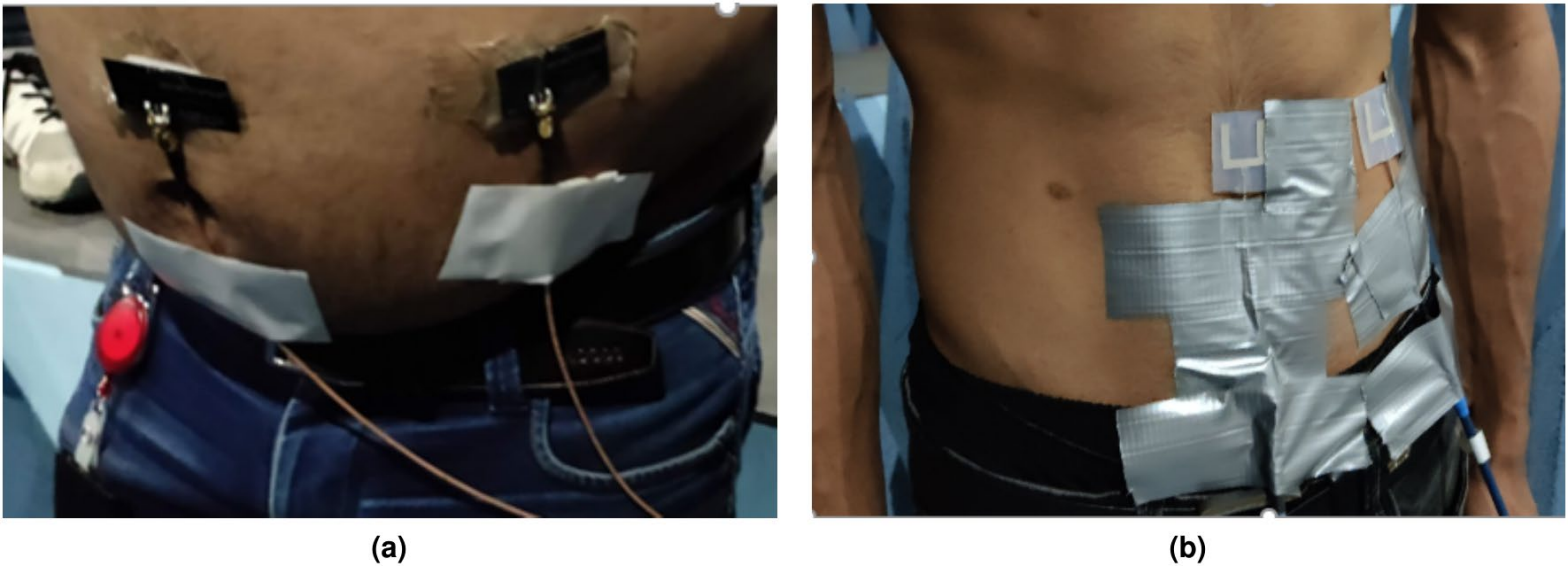
Measurement setup in an Anechoic chamber by using BLE and U-shaped Fat-IBC antennas by placing side-to-side (S2S). (**a**) On obese, and (**b**) on athletic human volunteers.

**Fig. 11:**
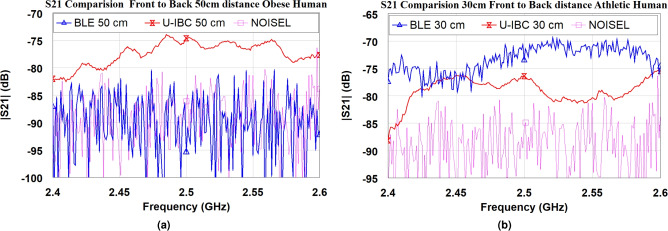
Measured transmission properties by using BLE and U-shaped Fat-IBC antennas. (**a**) Obese, and (**b**) athletic human volunteers at different distances.

**Table 4:** Comparative study between the transmission performance of BLE and U-shaped antenna-based Fat-IBC system at different distances over the human volunteer trials at 2.45 GHz.

Volunteers	Antenna type	Placement distance (cm)	$|S_{21}|$ (dB)	$\Delta |S_{21}|$ (dB)
Obese human	BLE antenna	10	−57.72	–
		20	−67.51	
		30	−74.91	–
		50	−89.83	–
		10	−37.12	+ 20.60
Obese Human	U-shaped antenna	20	−53.21	+ 14.30
		30	−64.35	+ 10.56
		50	−77.13	+12.17
		10	−58.68	–
Athletic Human	BLE antenna	20	−66.65	–
		30	−73.21	–
		10	−38.46	+ 20.22
Athletic Human	U-shaped antenna	20	−53.51	+ 13.14
		30	−77.21	−4.00

**Fig. 12:**
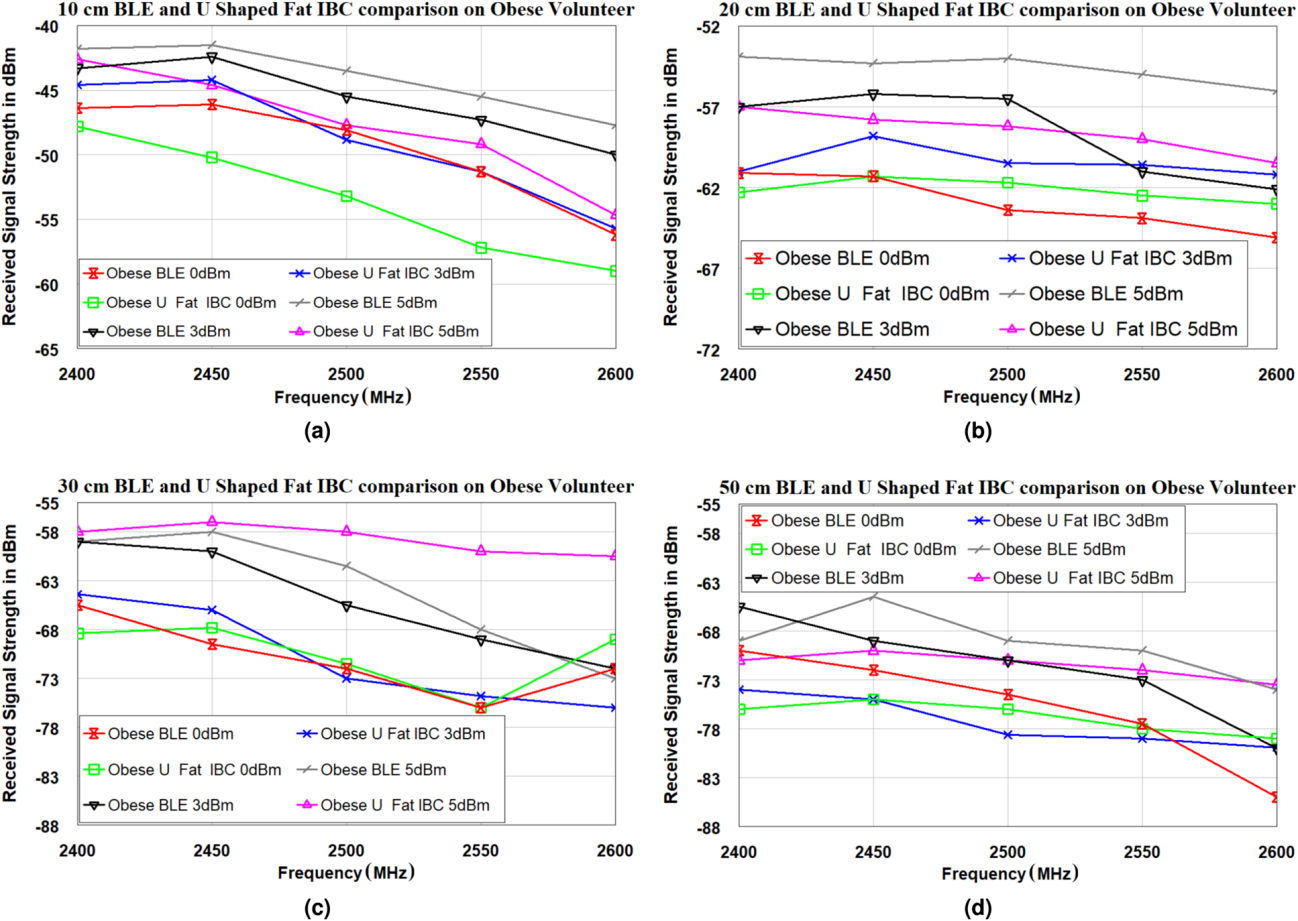
Characteristics of the signal strength for the U-shaped antenna on an obese volunteer at different positions. (**a**) 10 cm, (**b**) 20 cm, (**c**) 30 cm, and (**d**) 50 cm.

In this study, transmission coefficients ($|S_{21}|$) were measured using both BLE antennas and U-shaped Fat-IBC antennas within torso phantoms. These phantoms were modeled to represent two specific body types: an obese abdominal phantom and an athletic thoracic cavity phantom, with detailed fabrication methodologies elaborated in the reported literature^40,41^. The measurement setup, incorporating both torso phantoms within a small-scale anechoic chamber, is illustrated in Fig. 8a and b, respectively. In Fig.8a, the experimental configuration to compare the transmission characteristics of BLE antennas with U-shaped Fat-IBC antennas in obese torso phantoms is displayed. The phantom was positioned across the chamber’s internal separation wall in two distinct orientations: side-to-side and front-to-back (Fig. 8) over the phantom torso model. Fig.8b depicts a similar configuration for athletic torso phantoms, facilitating a comparative analysis of $|S_{21}|$ characteristics in both torso types. For these measurements, paired BLE chip antennas and U-shaped Fat-IBC antennas were tested using an N9918A FieldFox Microwave Analyzer. Data were recorded over a frequency range from 2 to 3 GHz, with five averages and 2% smoothing for enhanced signal clarity, and shown in Fig. 9. A high-resolution measurement was achieved by considering 1001 linearly spaced points per frequency sweep. Table 3 provides a comparative summary of the measured $|S_{21}|$ results for both BLE and U-shaped antennas, distinguishing signal variations across different torso environments and placement positions. The improvement in transmission coefficient ($\Delta |S_{21}|$) is also computed with the use of the proposed U-shaped antenna compared to the BLE antenna, as $\Delta |S_{21}|$ = $|S_{21}|$ BLE - $|S_{21}|$ U-IBC (dB), where a positive value indicates the U-shaped antenna outperforms BLE. Whereas, negative $\Delta |S_{21}|$ indicates the BLE outperforms the U-shaped antenna, attributed to the thinner fat layer in the athletic phantom. This comparison underscores the effectiveness of the Fat-IBC antenna over BLE in terms of transmission efficiency within diverse tissue compositions.

Furthermore, an in-depth comparative analysis of transmission characteristics derived from experimental trials on human volunteers of varying body mass index (BMI) levels using BLE and Fat-IBC U-shaped antennas is performed. Key performance parameters, including transmission coefficient and signal strength metrics, were measured to assess the impact of body composition on electromagnetic wave transmission. All measurements were conducted within a large anechoic chamber to eliminate external RF interference, thereby ensuring high precision. The selection of volunteers based on BMI, a primary indicator of body fat, allows for targeted investigation into how fat content influences electromagnetic propagation through biological tissues. Volunteers were classified into two distinct BMI categories: high BMI and average BMI, facilitating a comparative evaluation across diverse body types. Ethical clearance for these trials was granted by the Swedish Ethical Review Authority under the LIFESEC project (“Secure Data Communication in Human Tissues,” Approval No. 2019-01811). Two male volunteers with detailed anthropometric profiles participated: the first, representing a high-BMI group, was categorized as obese (BMI: 33.5, age: 36), while the second volunteer, with a BMI of 21 and an athletic build (age: 25), represented the average-BMI category. This selection enabled a comprehensive assessment of BLE and Fat-IBC antenna performance under realistic physiological conditions, highlighting the differences in transmission properties based on body fat content.

**Fig. 13:**
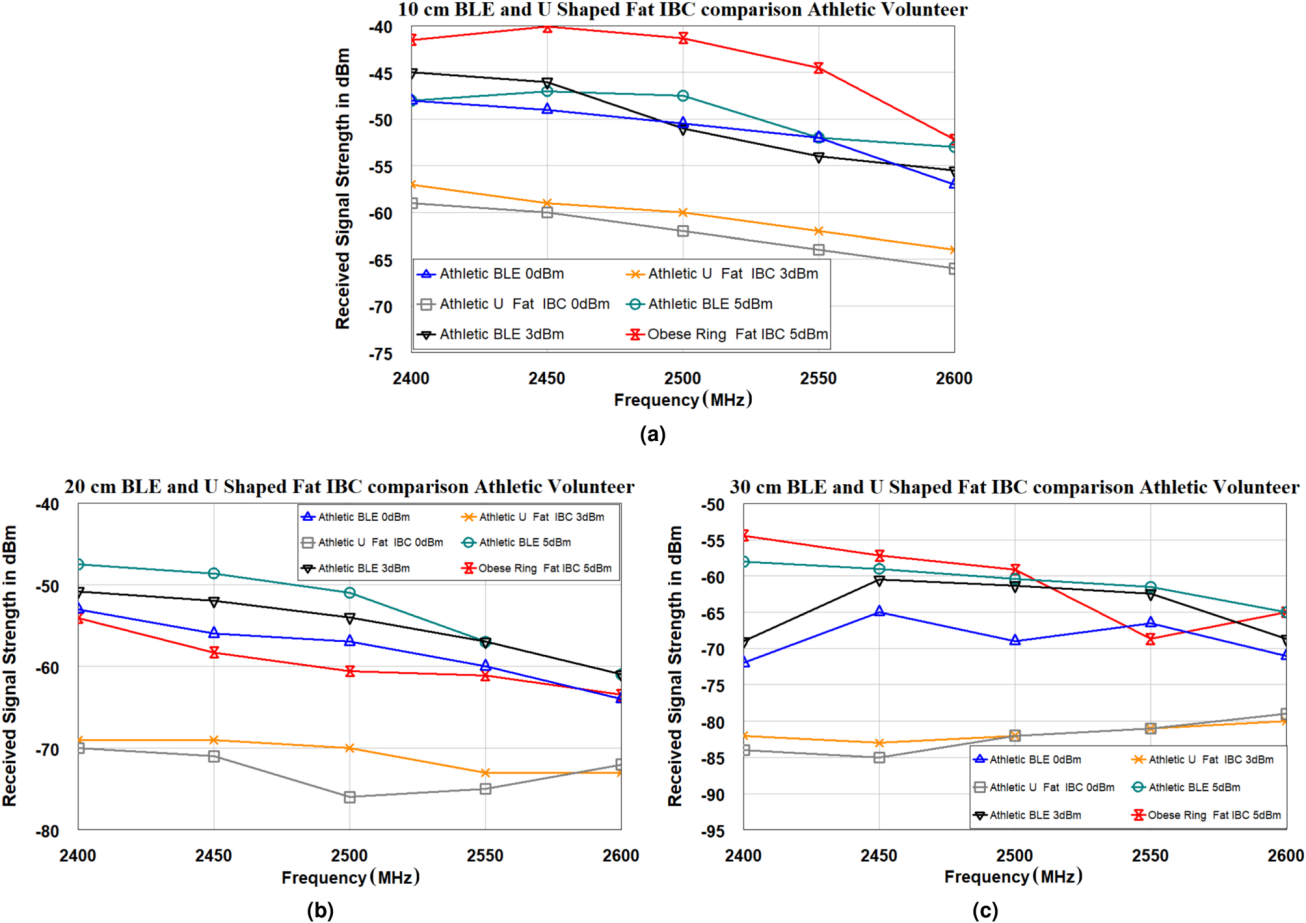
Signal strength properties were obtained from the use of BLE and U-shaped antenna on an athletic volunteer at different positions. (**a**) 10 cm, (**b**) 20 cm, and (**c**) 30 cm.

For volunteer-based trials evaluating transmission properties, the methodology closely resembles that used in the torso phantom measurements. The BLE and U-shaped Fat-IBC antennas were placed on the human body (specifically 10, 20, 30, and 50 cm apart) to measure the amplitude of $|S_{21}|$. The experimental setups for obese and athletic volunteers are presented in Fig. 10a and b, respectively. The measurement results allow for a comparative analysis of transmission performance across different body compositions. Fig.11a and b display the measured property of transmission coefficient for both the BLE and Fat-IBC antennas in the volunteer trials, showing clear performance differences between body types. A detailed summary of these findings is illustrated in Table 4. The results indicate that the U-shaped Fat-IBC antenna consistently achieves higher $|S_{21}|$ amplitude values than the BLE antenna for an obese human volunteer. It can also be seen from Table 3 and 4 that in the case of athletic phantom and athletic human, the BLE antenna provides better transmission gain compared to the proposed U-shaped antenna. The U-shaped antenna shows -77.21 dB compared to -73.21 dB for BLE at 30 cm on the athletic volunteer. This reversal is attributed to the thinner fat layer in the athletic subject, which reduces the fat-channel advantage at longer distances. The BLE antenna, being a general-purpose radiator not optimized for fat-layer coupling, is less sensitive to fat-layer thickness variations. The U-shaped antenna generally demonstrates superior performance, particularly in configurations with thicker fat layers and at the target operating frequency. This enhanced performance highlights the Fat-IBC system’s capacity to leverage the low-loss properties of body fat, optimizing signal integrity for intra-body communication applications.

**Table 5:** Comparative Evaluation of the Proposed Fat-IBC System with Existing State-of-the-Art Techniques.

References	In/On/Off Frequency Methodology body system Hardware				Lateral size of Tx/Rx$mm \times mm$	Biomedical environment	Gain of (Tx/Rx) Element	Transfer distance (cm)	$|S_{21}|$ (dB)/ Data rate (Mb/s)
^11^ [2019]	1-100 MHz	GC-BCC	In-body	180 nm CMOS	Tx = $0.89 \times 0.68$Rx = $1.45 \times 0.45$	Porcine	NA	10	−/100
^15^ [2019]	1.2 MHz	Ultrasound	In-body	Piezo crystals (X2)	$\pi \times 1^2$	Bone phantom	NA	8	−/0.20
^28^ [2023]	2.40-2.45 GHz	Fat-IBC	In-body	Off-the-self	$\pi \times 90.5^2$	Phantom tissues	NG	30	−61.41/92
^29^ [2025]	2.40-2.48 GHz	Fat-IBC (Resonator)	On-body	Off-the-self	$28 \times 28$	Three layer phantom	−9.62 dB	20	−52.41/92.4
^30^ [2025]	2.40-2.48 GHz	Fat-IBC (Antenna)	On-body	Off-the-self	$32 \times 32$	Three layer phantoms	−8.4 dB	20	−42.7/93
^42^ [2009]	1-30 MHz	CC-BCC	On-body	130 nm CMOS	$1 \times 1$	Human body	NA	–	−/8.5
This Work	2.40-2.48 GHz	Fat-IBC U-shaped (Antenna)	On-body	Off-the-self	$20 \times 20$	Torso and Human volunteer	−6.04 dB and −7.02 dB	30 (S2S) 30 (S2S) 30 (S2S) 30 (S2S)	−53.77 (OP) /− −69.76 (AP) /− −64.35 (OH) /− −77.21 (AH) /−

GC: Galvanically Coupled; CC: Capacitive Coupled; BCC: Body Channel Communication; NA: Not applicable; NG: Not given; OP: Obese phantom; AP: Athletic phantom; OH: Obese human; AH: Athletic human

To further analyze the performance of the Fat-IBC system, a network analyzer was configured in spectrum analyzer mode to accurately measure the strength of the received signal at the Rx end, with transmissions originating from the Tx antenna. The BLE and U-shaped Fat-IBC antennas were placed on the bodies of volunteers at various separation distances, as shown in Fig. 10. The microwave source MG3690A operated at three different power levels (0 dBm, 3 dBm, and 5 dBm) with sweep frequencies of 2400 MHz, 2450 MHz, 2500 MHz, 2550 MHz, and 2600 MHz. Signal strength measurements obtained for the BLE and U-shaped antennas are displayed in Fig. 12 for the obese volunteer and in Fig. 13 for the athletic volunteer, across varying antenna separations. The results consistently demonstrate that the U-shaped antenna delivers superior signal strength over BLE antennas at all tested distances. For trials involving the athletic volunteer, measurements at a 50 cm distance were omitted due to the volunteer’s slimmer body composition, which made maintaining consistent antenna placement challenging. It is perceived from the signal strength measurements (Figs. 12 and 13) that in some cases the BLE antenna shows higher received signal strength than the U-shaped Fat-IBC antenna at specific scenario and frequency points. These findings are attributed to: (i) the broadband impedance matching of the UWB ceramic BLE chip antenna, which maintains relatively flat performance across the 2.4-2.485 GHz range, whereas the U-shaped antenna is optimized for 2.45 GHz and its matching degrades at band edges; (ii) constructive multipath interference from body-surface waves at specific frequencies that may occasionally favor the BLE antenna’s omnidirectional radiation pattern; and (iii) at longer distances (30-50 cm) where received power approaches the noise floor, frequency-specific fluctuations become more pronounced and less indicative of the overall system trend. These findings highlight the Fat-IBC system’s enhanced transmission efficacy, establishing its suitability for high-reliability biomedical applications when compared to conventional BLE systems. This experimental data is crucial, offering valuable insights into Fat-IBC performance under realistic operational conditions and illustrating the effects of tissue composition and environmental factors on system performance.

Finally, a detailed comparative analysis of the proposed U-shaped antenna-enabled Fat-IBC system with conventional and antenna-based body channel communication (BCC) techniques available in the literature^11,15,28–30,42^ is summarized in Table 5. Conventional BCC approaches generally operate at low baseband frequencies and primarily utilize conductive or capacitive coupling mechanisms for signal transmission through the human body^11,15,42^. In contrast, the proposed Fat-IBC system exploits microwave-frequency operation, where the subcutaneous fat layer acts as a low-loss dielectric waveguide for electromagnetic propagation. Owing to this distinct propagation mechanism, the proposed system achieves improved transmission efficiency with comparatively lower path loss characteristics.

## Conclusion

This study evaluates the feasibility and performance analysis of a Fat-IBC system, utilizing compact and flexible U-shaped wearable antennas optimized for reliable, low-power intra-body communication. The proposed U-shaped wearable antenna is designed to operate at 2.45 GHz and encapsulated in a biocompatible PDMS layer. The designed antenna leverages the low-loss characteristics of adipose tissue to facilitate efficient signal propagation. Through both simulations and measurements on the torso phantoms of obese and athletic along with human volunteers, the Fat-IBC system demonstrated stable transmission over various distances, showcasing high-quality intra-body communication. The U-shaped Fat-IBC antennas exhibited superior signal stability and better performance compared to the BLE antenna, especially in adipose tissue. The BLE technology, common for low-power communication, experiences notable signal attenuation in body tissues. The optimized design of the U-shaped-based Fat-IBC system not only provides better performance but also ensures compliance with IEEE safety standards, as SAR levels were maintained well below the allowed limits. This establishes Fat-IBC as a promising alternative for continuous health monitoring and medical telemetry, with potential for further applications in biomedical fields. Moreover, the human trials involved only two volunteers (one obese and one athletic), which limits statistical analysis.

It is acknowledged that the human volunteer trials involved only two participants (one obese, one athletic), which restricts the statistical generalizability of the results. Inter-subject variability in fat layer thickness, tissue dielectric properties, body geometry, and skin moisture could all influence measured transmission coefficients. The observed performance reversal in the athletic subject at longer distances (where the BLE marginally outperformed the U-shaped antenna at 30 cm) exemplifies such sensitivity to fat-layer thickness. Future studies with a larger and more diverse unit, including participants with a range of BMI values and anatomical profiles, are planned to establish statistically robust performance bounds and to better quantify the relationship between adipose tissue thickness and Fat-IBC channel gain.

## Data Availability

The data sets generated and analyzed during the current study are available from the corresponding author on a reasonable request.
